# Supplementary material for: CXXC5 mediates growth plate senescence and is a target for enhancement of longitudinal bone growth
Source: Life Sci Alliance. 2019 Apr 10;2(2):e201800254. doi: 10.26508/lsa.201800254 (PMC6458850; doi:10.26508/lsa.201800254)
Supplement: Supplementary file 1 [file LSA-2018-00254_TableS1.docx]

**Table S1. Summary of high-throughput screening results.**

| **Category** | **Parameter** | **Description** |
| --- | --- | --- |
| Assay | Type of assay | *In vitro* binding assay |
|  | Target | CXXC5–DVL interaction |
|  | Primary measurement | Fluorescence intensity |
|  | Key reagents | FITC-tagged PTD-DBM peptide |
|  | Assay protocol | The protocol was provided in "Small molecule  inhibitors of the Dishevelled-CXXC5 interaction  are new drug candidates for bone anabolic  osteoporosis therapy" of Materials and Methods section |
| Library | Library size | 2280 compounds assayed in 96-well plates  as single compouns at 10 mM in DMSO |
|  | Library composition | Small molecules |
|  | Source | ChemDiv and Sigma LOPAC 1280 |
| Screen | Format | 96-well black polystyrene plates |
|  | Concentration(s) tested | Constant 30 μM concentration, 0.3% DMSO |
|  | Plate controls | DMSO-treated group |
|  | Reagent/ compound dispensing system | Reagents and compounds were dispensed manually |
|  | Detection instrument and software | FLUOstar OPTIMA (BMG LABTECH) |
|  | Assay validation/QC | Z-factor > 0.7 |
|  | Correction factors | N/A |
|  | Normalization | The sample result was normalized to positive control and is represented as % CXXC5–DVL interaction. |
| Post-HTS anaylsis | Hit criteria | < 10% inhibition |
|  | Hit rate | 1% |
