# Supplementary material for: CXXC5 mediates growth plate senescence and is a target for enhancement of longitudinal bone growth
Source: Life Sci Alliance. 2019 Apr 10;2(2):e201800254. doi: 10.26508/lsa.201800254 (PMC6458850; doi:10.26508/lsa.201800254)
Supplement: Supplementary file 2 [file LSA-2018-00254_TableS2.docx]

**Table S2. List of top-ranked positive lead compounds screened through an *in vitro* binding assay of chemical libraries including 2,280 small molecules.**

| **Compound** | **Structure** | **Empirical  Formula** | **CXXC5-DVL  interaction (%)** |
| --- | --- | --- | --- |
| 1 | 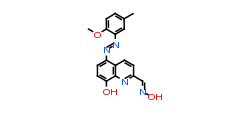 | C_18_H_16_N_4_O_3_ | 7.62 |
| 2 | 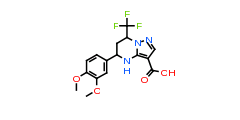 | C_16_H_16_F_3_N_3_O_4_ | 1.01 |
| 3 | 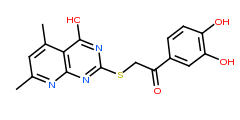 | C_17_H_15_N_3_O_4_S | 4.01 |
| 4 | 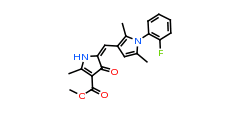 | C_20_H_19_FN_2_O_3_ | 4.76 |
| 5 | 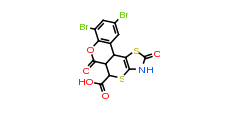 | C_14_H_7_Br_2_NO_5_S_2_ | 1.19 |
| 6 | 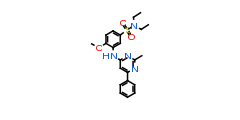 | C_22_H_26_N_4_O_3_S | 7.63 |
| 7 | 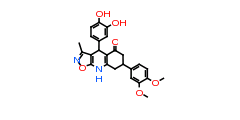 | C_25_H_24_N_2_O_6_ | 0.72 |
| 8 | 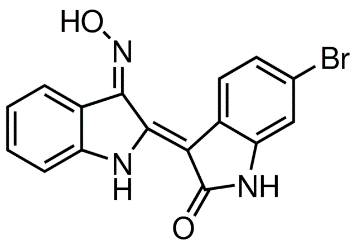 | C_16_H_10_BrN_3_O_2_ | 0 |
| 9 | 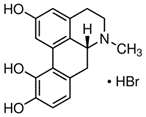 | C_17_H_17_NO_3_ · HBr | 2.5 |
| 10 | 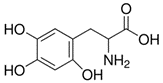 | C_9_H_11_NO_5_ | 1.39 |
| 11 | 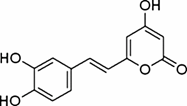 | C_13_H_10_O_5_ | 1.73 |
| 12 | 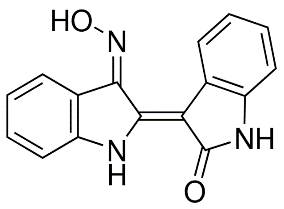 | C_16_H_11_N_3_O_2_ | 7.65 |
| 13 | 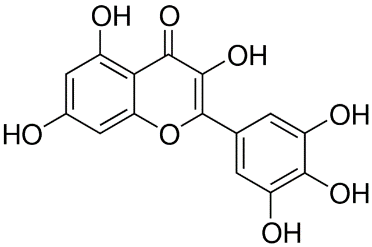 | C_15_H_10_O_8_ | 7.66 |
| 14 | 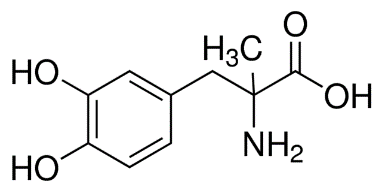 | C_10_H_13_NO_4_ | 7.65 |
| 15 | 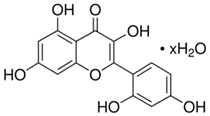 | C_15_H_10_O_7_ · xH_2_O | 7.43 |
| 16 | 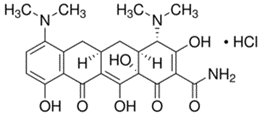 | C_23_H_27_N_3_O_7_ · HCl | 4.37 |
| 17 | 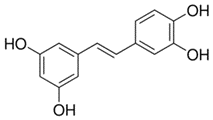 | C_14_H_12_O_4_ | 7.61 |
| 18 | 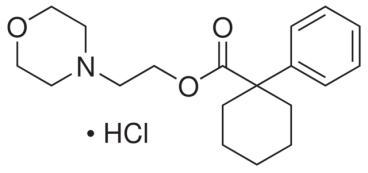 | C_19_H_27_NO_3_ · HCl | 5.7 |
| 19 | 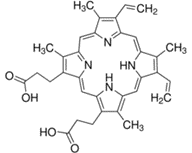 | C_34_H_34_N_4_O_4_ | 2.47 |
