# Supplementary material for: CXXC5 mediates growth plate senescence and is a target for enhancement of longitudinal bone growth
Source: Life Sci Alliance. 2019 Apr 10;2(2):e201800254. doi: 10.26508/lsa.201800254 (PMC6458850; doi:10.26508/lsa.201800254)
Supplement: Supplementary file 3 [file LSA-2018-00254_TableS3.docx]

**Table S3. Pharmacokinetic profiles for KY19382.**

| **PK Parameters** | **IV, 1 mg/kg** | | **IP, 5 mg/kg** | |
| --- | --- | --- | --- | --- |
|  | **mean** | **SD** | **mean** | **SD** |
| **t_max_** (hr) | N/A | - | 1.00 | 0.00 |
| **C_max_** (ng/mL) | N/A | - | 463.37 | 29.41 |
| **AUC_last_** (ng∙hr/mL) | 7832.81 | 651.28 | 6555.79 | 572.85 |
| **CL** (L/hr/kg) | 0.12 | 0.01 | 0.47 | 0.03 |
| **V_ss_** (L/kg) | 0.33 | 0.07 | N/A |  |
| **t_1/2_** (hr) | 3.33 | 1.34 | 16.20 | 3.86 |
| **F** (%) | N/A | - | 16.74 | - |

Pharmacokinetic parameters were based on the mean plasma concentration-time profiles of SD male rat (n=3). Pharmacokinetic parameters were obtained by non-compartmental analysis of the plasma concentration-time profiles using KineticaTM 4.4.1 (Thermo Fisher Scientific, Inc., Woburn, MA, USA). AUC_last_ was calculated from 0 to 24 hour. IV, intravenous; IP, intraperitoneal; T_max_, Time to maximum plasm concentration; C_max_, Maximum plasma concentration after intraperitoneal injection; AUC, Area under the curve; CL, clearance; V_ss_, Volume of distribution at steady state; T_1/2_, Elimination half-life; F, bioavailability.
