# Supplementary material for: CXXC5 mediates growth plate senescence and is a target for enhancement of longitudinal bone growth
Source: Life Sci Alliance. 2019 Apr 10;2(2):e201800254. doi: 10.26508/lsa.201800254 (PMC6458850; doi:10.26508/lsa.201800254)
Supplement: Supplementary file 4 [file LSA-2018-00254_Supplementary_Materials_and_Methods.docx]

**Supplementary Materials and Methods**

**Screening for compounds that inhibit the CXXC5–DVL interaction**

To initially identify small molecules that inhibited the CXXC5–DVL interaction, chemical libraries (2,280 compounds: 1,000 from ChemDiv and 1,280 from SigmaLOPAC) were screened by *in vitro* binding assay that was previously described (Kim et al, 2016). Briefly, 5 mg/ml purified DVL PDZ domain was incubated in each well of a 96-well Maxibinding Immunoplate (SPL) at 4°C for 16 hours. After the addition of 10 μM FITC-tagged PTD-DBMP to each well, each compound in the chemical library or control (DMSO) was treated to the well at a final concentration of 30 μM. The fluorescence intensity was measured using a Fluorstar Optima microplate reader (BGM Lab Technologies, Ortenberg, Germany). The binding values were calculated as a percent ratio of the fluorescent intensity normalized to the DMSO-treated control. Nineteen compounds exhibited lower than 10% for the CXXC5–DVL interaction. Among these compounds, indirubin analogs including BIO and I3O were identified. A summary of the high-throughput screening results is provided in Table S1.

***In silico* docking (Flexible docking)**

NMR structure of DVL PDZ domain with known ligand, sulindac, was obtained from the Protein Data Bank (PDB code: 2KAW). BIO, I3O, and KY19382 were docked to the sulindac binding site of PDZ domain by using Flexible docking method in Discovery Studio software adopting the CHARMm force field (Dassault Systèmes BIOVIA, Discovery Studio Modeling Environment, Release 2017, San Diego: Dassault Systèmes, 2016). For the docking analysis, active site was defined within 10.6 Å sphere centered from the ligand and the core site was redefined including residues I264, S265, I266, L321, R324, and V325, known as make up the receptor-ligand interaction. Based on the docking results, various scoring functions (Ligscore1_Dreiding, Ligscore2_Dreiding, PLP1, PLP2, PMF, DOCKSCORE) were determined to calculate binding energy the most predictive binding mode.

**
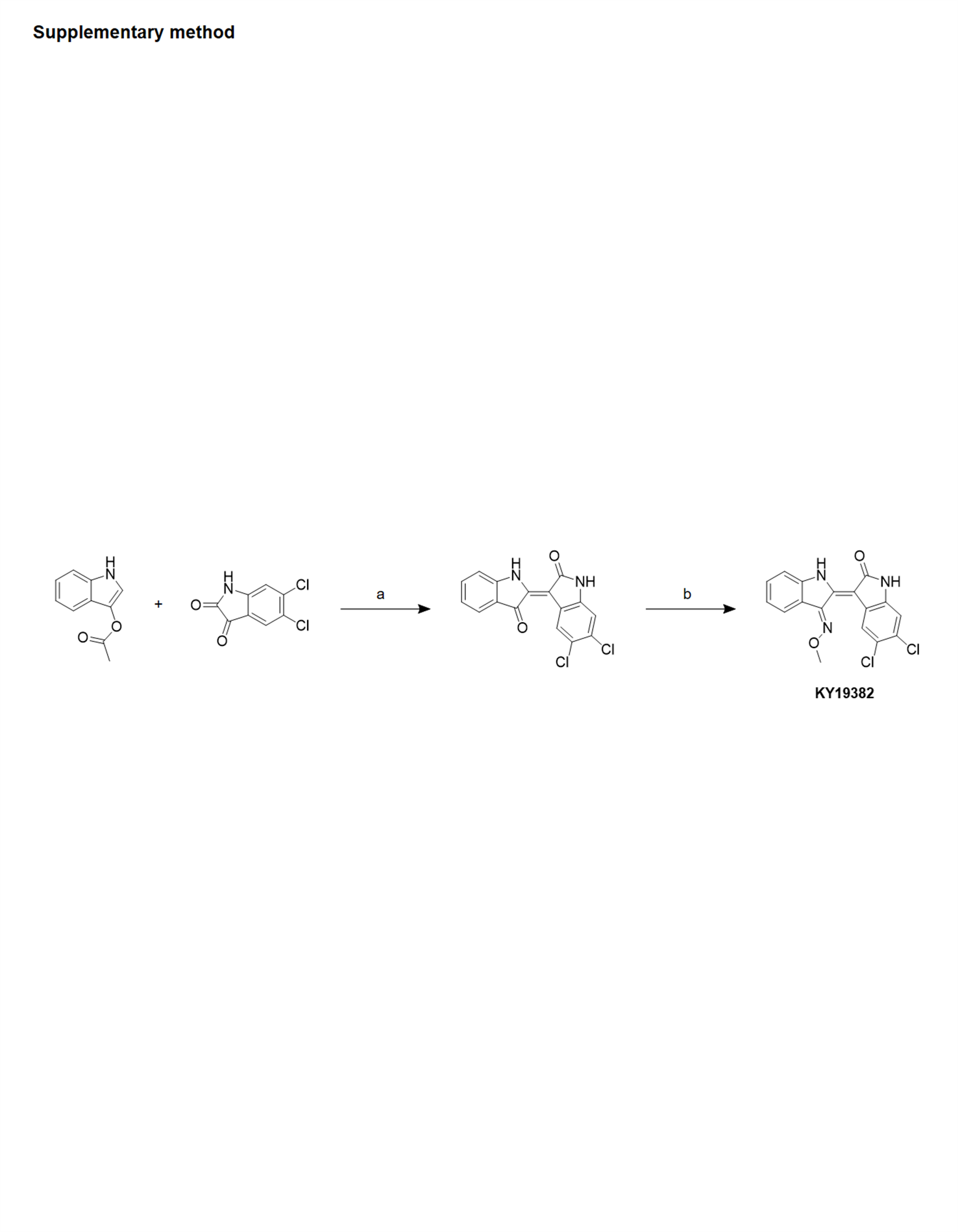
A synthetic procedure of KY19382.**

Scheme 1. Reagent: (a) Na_2_CO_3_, MeOH, reflux; (b) H_2_NOCH_3_·HCl, pyridine, reflux.

To a solution of 3-acetoxyindole (405 mg, 2.32 mmol) in methanol (0.025 M), 5,6-dichloroisatin (500 mg, 2.32 mmol) and sodium carbonate (613 mg, 5.79 mmol) were added. The reaction mixture was refluxed for overnight. The dark precipitate was filtered and washed with aqueous ethanol and recrystallized by ethanol and H_2_O solution (1:1 (v/v)). The desired compound, 5,6-dichloro-indirubin was dried *in vacuo* to give as a purple solid with 63% yield. ^1^H NMR (400 MHz, DMSO-*d6*) δ 11.10 (s, 2H), 8.89 (s, 1H), 10.70 (s, 1H), 7.63 (d, 1H, *J* = 8.0 Hz), 7.60-7.55 (m, 1H), 7.40 (d, 1H, *J* = 8.0 Hz), 7.05-7.01 (m, 1H). Next, to a solution of 5,6-dichloro-indirubin (200 mg, 0.61 mmol) in pyridine (0.15 M) was added the anhydrous hydroxylamine hydrochloride (509 mg, 6.1 mmol). The reaction mixture was refluxed (120°C) for overnight. After the reaction was completed, the solvent was evaporated under reduced pressure and the residue was fully dissolved in ethyl acetate and water with sonication. The reaction mixture was extracted using ethyl acetate (100 ml × 2) and washed with saturated aqueous sodium bicarbonate solution (200 ml), dried over anhydrous MgSO_4_. The crude product was recrystallized with methanol and hexane solution, and the desired product, 5,6-dichloroindirubin-3`-methoxime was dried *in vacuo* to give as a reddish brown solid with 31% yield. ^1^H NMR (400 MHz, DMSO-*d6*) δ 11.63 (s, 1H), 10.92 (s, 1H), 8.73 (s, 1H), 8.02 (d, 1H, J = 4.0 Hz), 7.41-7.37 (m, 2H), 7.02-6.98 (m, 1H), 6.94 (s, 1H), 4.33 (s, 3H).

***In vivo* pharmacokinetics of KY19382**

Animal studies were approved by the Institutional Animal Care and use committee at KRIBB. Briefly, specific pathogen-free male Sprague−Dawley (SD) rats (10 weeks old, body weight 298−315 g), purchased from Koatech Co. (Kyeonggi, Republic of Korea), were given a single dose of KY19382 intravenously (iv, 1 mg/kg, n = 3) or intraperitoneal (ip, 5 mg/kg, n = 3). Dosing solutions were prepared in dimethylacetamide (DMAC)/polyethylene glycol 400 (PEG400) (20/80, v/v %) for administrations, and administered at dosing volumes of 5 and 10 ml/kg for iv and ip, respectively. A 100 μl aliquot of each plasma sample was prepared, and three volumes of ice-cold acetonitrile containing carbamazepine (internal standard) were added. The mixture was centrifuged at 910 g for 10 minutes, and the supernatant was subjected to LC−MS/MS analysis. Pharmacokinetic parameters were calculated by standard noncompartmental analysis of plasma concentration−time profiles using Kinetica 4.4.1 (Thermo Fisher Scientific, Inc., Woburn, MA, USA). The areas under the plasma concentration−time curves (AUC) were calculated by the linear−trapezoidal method. Systemic plasma clearance (CL_P_) was calculated as follows: CL_P_ = dose/AUC_inf_. Terminal elimination half-life (t_1/2_) was calculated by the following equation: t_1/2_ = 0.693/λ_Z_ where λ_Z_ is the terminal disposition rate constant. Volume of distribution at steady state (V_SS_) was calculated as follows: V_SS_ = dose × AUMC_inf_/(AUC_inf_)^2^, where AUMC_inf_ is the area under the first moment of the plasma concentration−time curve extrapolated to infinity. Bioavailability (F) was calculated as follows: F (%) = (AUC_ip_/AUC_iv_)(dose_iv_/dose_ip_) × 100.
